# Could Metformin Manage Gestational Diabetes Mellitus instead of Insulin?

**DOI:** 10.1155/2016/3480629

**Published:** 2016-08-14

**Authors:** Hend S. Saleh, Walid A. Abdelsalam, Hala E. Mowafy, Azza A. Abd ElHameid

**Affiliations:** Obstetrics and Gynecology Department, Faculty of Medicine, Zagazig University, Zagazig 44519, Egypt

## Abstract

Gestational diabetes mellitus (GDM) complicates a significant number of pregnancies. Blood glucose control improves perinatal outcomes. Medical nutrition therapy is the foundation in management.* Aim of This Study*. To evaluate efficacy of metformin in comparison to insulin for managing GDM.* Methods.* In prospective randomized comparative study, 150 antenatal women whose pregnancies had been complicated by GDM and did not respond to diet alone were recruited from antenatal clinics at Obstetrics Department in Zagazig University Hospitals from November 2012 to December 2014. They were divided randomly into two groups, 75 patients in each, and were subjected to either insulin or metformin medication. Outcomes were comparing the effects of both medications on maternal glycemic control, antenatal complications, and neonatal outcome.* Results*. No significant difference in controlling high blood sugar in GDM with the use of metformin or insulin (*P* = 0.95, 0.15). Maternal complications in both groups had no significant difference and fetal outcomes were as well similar except the fact that the hypoglycemia occurred more in insulin group with *P* value 0.01.* Conclusion*. Glycaemic control in GDM can be achieved by using metformin orally without increasing risk of maternal hypoglycemia with satisfying neonatal outcome.

## 1. Introduction

 Gestational diabetes mellitus (GDM) is a condition with any level of glucose intolerance which began or was detected for first time during pregnancy despite type of management; it may also relate to situations that continue after pregnancy. It affects approximately 7% of pregnancies with an incidence of more than 200,000 cases per year [1].

40–60% of gestational diabetes mellitus (GDM) cases have chance of developing diabetes mellitus over the 5–10 years after pregnancy [2].

Older and more obese pregnant women have the highest incidence of GDM. It is associated with numerous undesirable outcomes over the short and long term for both mother and neonate [3]. Incidence of preeclampsia and rate of cesarean section increased in GDM as some of the short term complications. Developing type 2 diabetes (T2D) after pregnancy is one of the long term bad maternal outcomes [4]. Extreme mother-to-fetus glucose transmit is an augmented hazard for congenital defects, neonatal death, and still birth. The hyperglycemic environment intrauterine influences children later in life [5].

Macrosomia is an extraimportant complication which is a risk factor for instrumental delivery, shoulder dystocia, and cesarean section during delivery. Neonatal hypoglycemia directly after birth is one of the most risky complications, putting neonate in danger [6].

The first screening test for GDM was advised in 1973, in the form of the 1-h 50 gm oral glucose tolerance test [7]. Some guidelines recommended screening common screening to all pregnant women to improve pregnancy outcomes. Others excluded low risk patients who were <25 years old with normal body weight, no history of abnormal glucose metabolism, no first-degree relatives with diabetes, and no history of poor obstetric outcomes [8].

During first antenatal visit, pregnant women with high risk for GDM should be screened for it immediately. If negative, they should be retested between 24 and 28 weeks of gestation. Average risk pregnant women (neither high nor low risk) should be screened between 24 and 28 weeks of gestation [1].

The World Health Organization (WHO) recommends using a 75-gm glucose tolerance test for screening and diagnosis. The doorstep values are a fasting glucose concentration of more than 126 mg/dL (7.0 mmol/L) and/or a 2-h glucose concentration of more than 140 mg/dL (7.8 mmol/L) [9].

Recently, trials have exhibited that efficient management of hyperglycemia in women with GDM is the main principle to prevent hyperinsulinemia and fetal macrosomia [10].

We diagnosed GDM by the American Diabetes Association (ADA) criteria, depending on 75-gm glucose load then checking the fasting serum glucose concentration, 1-h glucose concentration, and 2-h glucose concentration [11].

The glucose threshold values are 95 mg/dL (5.3 mmol/L), 180 mg/dL (10.0 mmol/L), and 155 mg/dL (8.6 mmol/L), respectively. Two or more abnormal values are required for diagnosis. Some studies have shown that a single abnormal value is significantly associated with amplified neonatal hazards [12].

When the World Health Organization (WHO) advised using a 75-gm glucose tolerance test for screening and diagnosis of GDP with the threshold values of a fasting glucose concentration of more than 126 mg/dL (7.0 mmol/L) and/or a 2-h glucose concentration of more than 140 mg/dL (7.8 mmol/L), about twice as a lot of patients will be positive diagnosis [9].

The main management started by dietary and exercise counseling, but about 20–60% of GDM patients often require pharmacological treatment, which has conventionally been insulin [13].

Dietary adjustment is frequently called medical nutrition therapy. Evidence shows that it is efficient in glycaemic control and improving pregnancy and neonatal outcomes [14].

American Diabetes Association (ADA) suggests exercise programs in moderate level for those who have no medical or obstetrical complications, in the form of 3 or more times per week for 30 min [11].

The American College of Obstetrics and Gynecology (ACOG) recommended that GDM patients should keep up the following capillary blood glucose values: preprandial glucose <95 mg/dL (5.3 mmol/L), 1-h postprandial glucose 130–140 mg/dL (7.8 mmol/L), and 2-h postprandial glucose <120 mg/dL (6.7 mmol/L) [15].

Others recommend maintaining fasting glucose levels of <90–99 mg/dL (5.0–5.5 mmol/L), 1-h postprandial glucose levels of <140 mg/dL (7.8 mmol/L), and 2-h postprandial glucose levels of <120–127 mg/dL (6.7–7.1 mmol/L) [16].

There is agreement that measuring postprandial glucose levels is more important compared to preprandial levels since the former associates better with definite neonatal risks like hypoglycemia, macrosomia, and shoulder dystocia [17].

The pharmacological interference is in the form of either subcutaneous insulin which has been considered the standard for management of GDM or oral hypoglycaemic agents (metformin and glyburide) [18].

Insulin regimens frequently consist of intermediate acting insulin such as isophane and short acting agents such as regular recombinant insulin (Humulin R) [19].

Adjustments of its doses depend on the patient's body mass index, glucose levels, and lifestyle. Insulin therapy has several disadvantages including multiple daily injections, the risk of hypoglycemia, and maternal weight gain [20].

Health education for dose adjustment of insulin is essential to provide confident safe self-administration of insulin. Currently, considerable costs of health education on the safe use of insulin in addition to the cost of the drug itself are chased. Observably, oral therapy if safe and effective could be more satisfactory and desired [3].

So, it is good idea to use oral hypoglycemic agents in controlling blood sugar. Hypothetically, metformin is an alternative to insulin in the treatment of hyperglycemia during pregnancy. It reduces hyperglycemia by suppressing hepatic glucose output so it reduces hepatic gluconeogenesis and it is intensifying insulin sensitivity therefore enhancing peripheral glucose uptake [21].

It has been found to have a rate of maternofetal transfer of 10–16%. Before, it had not been widely used in GDM but, nowadays, growing studies focus on investigating the effectiveness and safety of metformin in such cases. These Studies were either case-control, observational trials or randomized controlled trials (RCTs). Still its use in pregnancy is controversy [22].

The aim of this study is to compare efficacy and safety of metformin to those of insulin on glycemic control and maternal and neonatal outcomes in GDM to reach end conclusion about the possibility of replacing insulin by metformin in pregnancy.

## 2. Methods

It is a prospective randomized comparative study. One hundred and fifty antenatal women whose pregnancies had been complicated by GDM and did not respond to diet modifications or nutritional instructions alone in 3 weeks were recruited from antenatal clinics at Obstetrics Department in Zagazig University Hospitals from November 2012 to December 2014. GDM was diagnosed at 26–34 weeks using WHO criteria: fasting plasma glucose ≥7.0 mmol/L or 2-h value >7.8 mmol/L following a 2-h 75 g OGTT [23].

Exclusion criteria were type 1 and type 2 diabetes and anyone who was already on insulin treatment, recognized fetal anomaly by ultrasound investigation, the fact that mother had hypersensitivity or intolerance to metformin intake like gastrointestinal side effects, liver or kidney diseases, and any obstetric high risk conditions. After the study protocol was approved by the Research Ethics Committee of the Zagazig University Hospitals, the research course was completely explained to the participants receiving their verbal and written informed consents. They were divided randomly into two groups by permuted block randomization. Each group had 75 pregnant mothers.* Group 1* received metformin orally initially at dose of 500 mg/day with meals which slowly increased up to 3000 mg in divided doses as tolerated by the patient and till controlled glycemic profile was realized. If the target was not achieved or tolerance was not achieved then insulin was commenced.

*Group 2* received insulin as a combination of short acting (Actrapid) and intermediate acting (Mixtard) human insulin as twice daily injections before breakfast and before dinner to face the three meals and three snacks per day depending on individual patient requirement, in order to achieve the desired glycemic goals. 24-hour total insulin dose was estimated using 0.6 units/kg body weight in 1st trimester, 0.7 units/kg body weight in 2nd trimester, 0.8 units/kg body weight from 28 to 32 weeks of gestation, 0.9 units/kg body weight from 32 to 36 weeks of gestation, and 1 unit/kg body weight from 36 weeks onwards. Monitoring at home was done by estimating blood glucose levels. Fasting and 2-hour postprandial blood sugar had been measured after the three main meals. The target of management was to maintain fasting blood sugar (FBS) at <100 mg/dL (5.5 mmol/lit) and postprandial blood sugar (PPBS) levels at <120 mg/dL (7 mmol/lit).

Glycemic profile, fasting blood sugar (FBS), and postprandial blood sugar (2 hr PPBS) were done weekly for all cases.

Dose modifications of drugs were made at each antenatal visit weekly till delivery. Usual obstetric care was offered at the antenatal clinics including ultrasound examination which was done at first visit (dating scan) and then at 16–19 weeks (anomaly scan) and then monthly after 28 weeks as fetal well-being scan. HbAIC was done at entrance of study and at around 37 weeks of pregnancy. Mode and time of delivery were decided around 38 weeks of pregnancy. Maternal outcome in the form of glycemic control, medical complications, and mode of delivery were documented. Neonatal outcomes were recorded and all were statistically analyzed.

The recorded data was evaluated using SPSS12.0. Mean with SD was reported for all continuous variables and was expressed as mean ± standard deviation (SD). Qualitative analysis was done using Student's *t*-test. Two-sample independent Student's* t*-test and Mann-Whitney test were used for continuous data. For quantitative analysis chi-square test Fisher Exact test, and Mann-Whitney test were used. Statistical significance was considered at *P* value of <0.05.

## 3. Results

**Figure 1:**
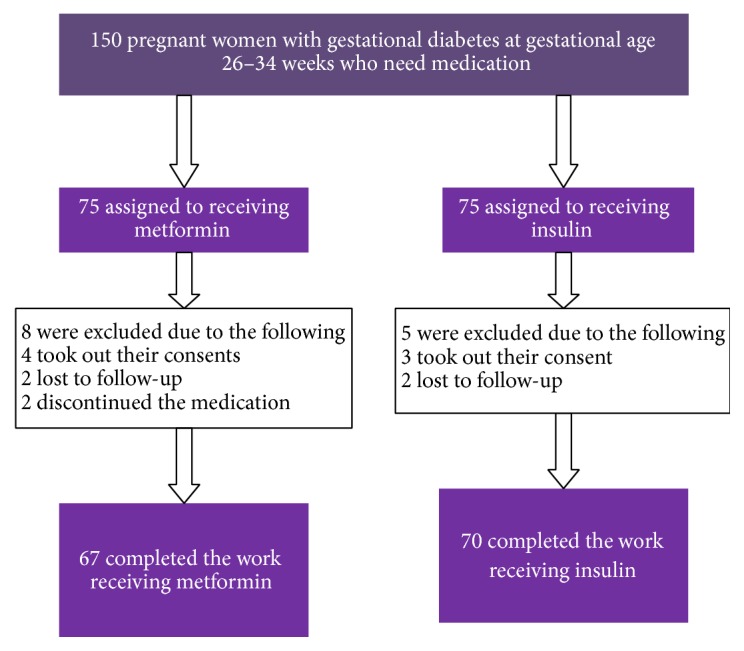
Study in flow chart.

*This prospective comparative study* is to compare the usefulness of metformin versus human insulin in patients with GDM. A total of 150 patients with GDM were registered in the study. They met the inclusion criteria and were randomized to treatment with metformin or insulin. 137 participants completed the study and their data was finally analyzed, with 67 patients in metformin group and 70 patients in insulin group. The design and subject course through the study are exemplified in Figure 1.

The demographic characteristics of metformin and insulin groups at the time of diagnosis of GDM were similar (Table 1).

Fasting and 2-hour postprandial blood glucose levels were statistically analogous in two groups. Glycemic targets were achieved and maintained throughout pregnancy in the intention variety with no statistical difference in both groups (Table 2).

GDM was diagnosed around the period of 26–34 weeks of gestation in our participants. Preeclampsia developed in 13 patients of the metformin group and in 12 patients of insulin group. Seven patients developed preterm labour in metformin group versus 5 patients in insulin group. Eight patients in metformin group developed polyhydramnios whereas only 6 patients in insulin group showed polyhydramnios on growth scan. Urinary tract infection was found in 4 patients in metformin groups versus 3 in insulin group. No significant difference was found between both groups according to medical disorders which developed during antenatal period (Table 3).

**Figure 2:**
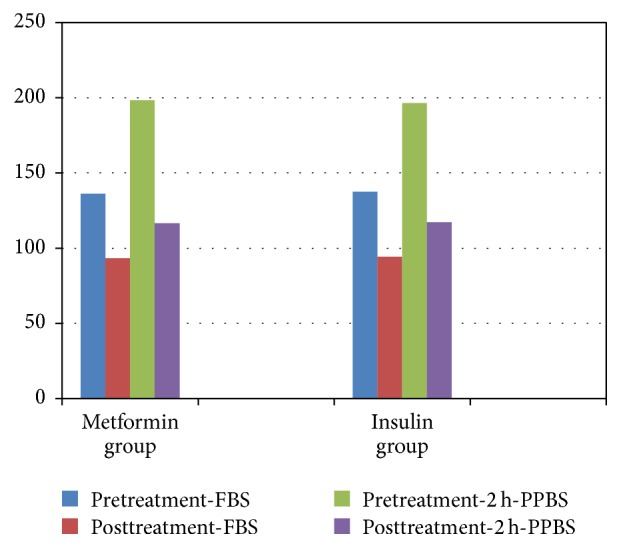
The figure shows pattern of blood sugar in both groups before and after treatment (fasting blood sugar (FBS) and 2 h postprandial blood sugar (2 h PPBS)).

**Figure 3:**
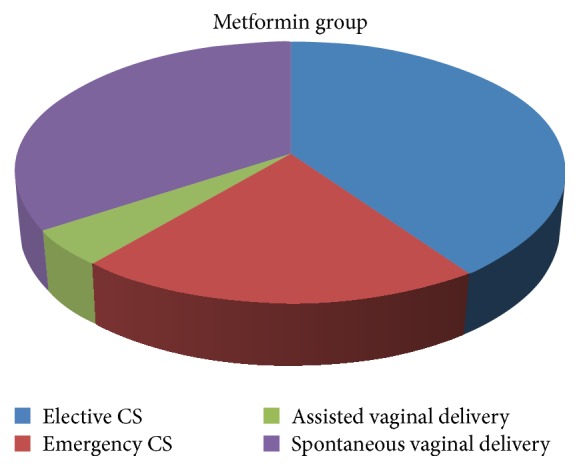
The figure shows % of modes of deliveries in metformin group.

**Figure 4:**
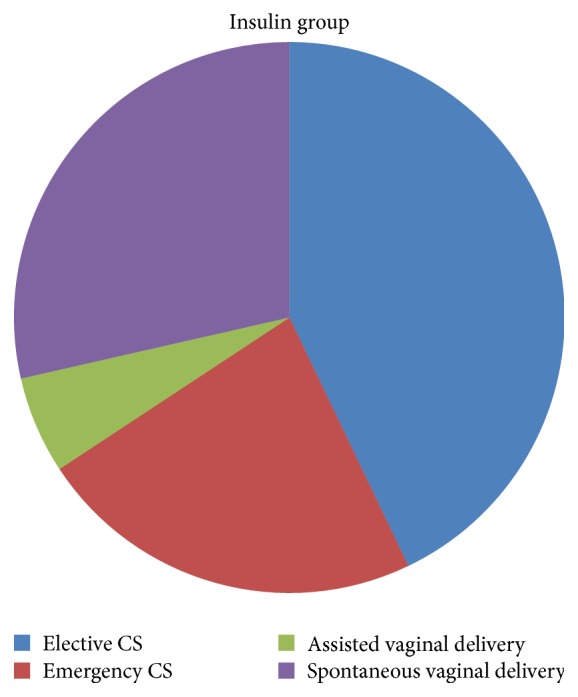
The figure shows % of modes of deliveries in insulin group.

As for mode of delivery, statistically, no significant differences were found between both groups as 40.2% of metformin group underwent elective cesarean section versus 42.8% in insulin group. 20.8% of metformin group underwent emergency cesarean section versus 22.8% in insulin group. 34.3% of metformin group underwent spontaneous vaginal delivery versus only 28.5% in the insulin group. Assisted vaginal delivery using ventouse was done in cases of metformin group and cases in the insulin group (Table 4) (Figures 2, 3, and 4).

Neonatal outcomes were presented in Tables 5(a) and 5(b). Occurrence of transient tachypnea, respiratory distress, neonatal jaundice, need for phototherapy, or admission to neonatal intensive care unit in both groups was comparable with no significant difference. Hypoglycemia developed in 7 babies of metformin group and 15 cases in insulin group with *P* value 0.01 which is statistically significant. No birth trauma happened in any baby of any group, Table 5(a). There was no significant difference between both groups with regard to mean gestational age at birth, Apgar score at 5, estimated fetal weight, or presence of congenital anomalies Table 5(b).

## 4. Discussion

Gestational diabetes mellitus (GDM) has been described as any extent of glucose intolerance with first detection throughout pregnancy and, depending on the diagnostic tests in employment, it complicated 1–14% of all pregnancies in current years [24].

It is one of the most common medical complications of pregnancy which is related to numerous adverse results to mother and raised risks of prenatal morbidity. So, the management of GDM seeks to diminish such risk of unfavorable neonatal and pregnancy complications [25].

Management is based on self-monitoring of blood glucose concentrations, diet, and physical exercise. When these measurements cannot control blood glucose levels in pregnant women, pharmacological therapy is needed to be added in addition [26].

Using of any medication during pregnancy is limited by its safety which depends on crossing the placenta and if it has effect on the fetus or not. A lot of drugs frequently used in pregnancy cross the placenta and may not exert effects on the fetus [27].

For a lot of years, conventionally, the first-line pharmacological management of GDM has been insulin with no fetal or neonatal obstacles [28].

The drawbacks of insulin are as follows; it needs health education, needs many daily subcutaneous injections, and requires dose modification depending on body mass index of patient, occurrence of hypoglycemia, and gaining weight in mother [14]. So, secure and valuable oral therapy would be more suitable and preferred by patients [29].

The controversy of using oral hypoglycemic agents like glyburide and metformin in pregnancy is related to concerns about their safety for the developing fetus [30]. The American College of Obstetricians and Gynecologists (ACOG) does not support or recommend against the use of oral antidiabetic agents in pregnancy [15]. But The United Kingdom National Institute for Health and Clinical Excellence (NICE) recommends metformin use before and during pregnancy and supports metformin and glyburide as choices for handling of gestational diabetes [2].

Most studies discussed the amount of transplacental passage of glyburide; Kraemer et al. recognized active transport of glyburide from the fetal circulation to the maternal one that may guard the fetus from contact with the drug [31].

Langer et al. could not detect any glyburide in the cord blood at delivery despite its presence in maternal serum [32].

Other studies indicate that fetal concentrations of glyburide may be 1% to 2% of maternal concentration [33].

Moore et al. had compared neonatal outcomes of pregnant women with GDM that were managed by metformin to those with glyburide in randomized study but found no significant difference [34].

In the current study, we preferred using metformin as one of the oral hypoglycemic medications. The occurrence of unfavorable outcomes either in pregnancy or in neonate were not raised in those who were managed with metformin compared with those who were managed with insulin except the fact that the neonatal hypoglycemia happened more in insulin group.

The results of our study to high extent were similar to studies by Coetzee and Jackson in 1970. They were the first researchers who studied metformin during pregnancy in women with insulin-independent diabetes. Their study had also two groups of patients; one received metformin and the second one received insulin. The maternal and perinatal outcome were the same for both [35].

The studies on this issue have been continuing from Coetzee and Jackson until Lim in 1997 [36], who was the first one that described that GDM can be managed efficiently and securely with oral hypoglycemic drugs with no distinction in pregnancy outcomes.

Then, in 2000, Hellmuth and colleagues [37] presented a cohort study of type 2 DM pregnant women on metformin in opposition to glyburide versus insulin. Their results proposed apprehension for the use of metformin because of the raised rate of preeclampsia (32% metformin versus 7% glyburide versus 10% insulin) and intrauterine fetal death (8% versus 0% versus 2.3%, correspondingly). Conversely, this study has become controversial with reviewers arguing that women in the study were not sound matched. Those women who received the metformin were morbidly obese and started to use the medication later on in the pregnancy. Consequently, the women were essentially at threat for poor pregnancy outcomes not related to metformin [38].

Rowan et al. 2008 [39] had randomized Australian study performed on women with gestational diabetes between 20 and 33 weeks of pregnancy getting metformin or insulin. There was no difference in efficacy between both groups in controlling glucose levels. Infants of metformin group had a lower rate of hypoglycemia compared with infants of insulin group with no more neonatal outcomes.

Glueck and his colleagues proved that, in many studies, metformin in pregnancy was not associated with increased incidence of medical disorders in pregnancy and mainly preeclampsia or hypoglycemia also associated with less spontaneous abortion, fetal anomalies, and neonatal complications [40].

The results of this current study were comparable to the findings of Glueck et al. as we also found that metformin intake during pregnancy was not associated with increasing rate of preeclampsia or neonatal complications.

Rai et al. [22] in their prospective observational study were comparing metformin to insulin for patients with GDM and type 2 DM (T2DM) in pregnancy. They found that glycemic control was better with metformin after 1 week of therapy and also throughout gestation compared to insulin and also found no major complications or perinatal deaths related to metformin uptake. They proved that metformin is clinically efficient, inexpensive, and a harmless alternative to insulin therapy in pregnant diabetic women.

These days, more studies center on investigating the effectiveness and safety of metformin when used during pregnancy in managing GDM. Some are observational studies and others are case-control trials [41]. The randomized controlled trials (RCTs) are present but with little samples which are deficient in the authority to represent valid conclusion about the use of metformin for managing GDM.

Our study had the same drawback that although it is randomized one, the sample size was small.

## 5. Conclusion

Metformin is effective and safe in gestational diabetes mellitus (GDM) and could be used instead of insulin in such cases.

## Figures and Tables

**Table 1:** Demographic profile of metformin and insulin groups.

Parameter	Metformin group *N*: 67	Insulin group *N*: 70	*P* value
Age (years)	31 ± 3.42	29.8 ± 2.18	0.398

Parity	3.05 ± 1.61	3.24 ± 1.72	0.253

Family history			
(i) Diabetes	40%	42%	0.911
(ii) Hypertension	31%	34%	0.897
(iii) Preeclampsia	29%	27%	0.963

Mean gestational age of diagnosis of GDM	27.28 ± 3.458	29.31 ± 3.12	0.348

BMI-early pregnancy (kg/m^2^)	30.52 ± 3.17	31.58 ± 30.12	0.614

BMI-late pregnancy (kg/m^2^)	34.28 ± 2.17	37.11 ± 3.87	0.016

**Table 2:** Fasting blood sugar (FBS) and mean 2-hr postprandial blood sugar at start and throughout treatment (mg/dL).

Parameter	Metformin group	Insulin group	*P* value
Mean fasting (FBS) (mg/dL) at starting of treatment	136.09 ± 39.85	137.56 ± 41.10	0.869
Mean 2-hr postprandial blood sugar at starting of treatment	198.32 ± 214.67	196.52 ± 15.45	0.451
Mean fasting (FBS) (mg/dL) throughout treatment	93.25 ± 13.7	94.33 ± 11.1	0.953
Mean 2-hr postprandial blood sugar throughout treatment	116.52 ± 3.53	117.12 ± 3.45	0.158

**Table 3:** Maternal complications in study groups.

Maternal complication	Metformin group		Insulin group		*P* value
	*N*: 67		*N*: 70		
	%	*N*	%	*N*	
Preeclampsia	19.4%	13	17.1%	12	0.273
Preterm	10.4%	7	7.1%	5	0.039
Polyhydramnios	11.9%	8	8.5%	6	0.710
Urinary tract infection	5.9%	4	4.2%	3	0.801

**Table 4:** Mode of delivery between metformin and insulin groups.

Mode of delivery	Metformin group		Insulin		*P* value
	*N*: 67		*N*: 70		
	%	*N*	%	*N*	
Elective LSCS	40.2%	27	42.8%	30	0.61
Emergency LSCS	20.8%	14	22.8%	16	0.37
Assisted vaginal delivery (vacuum extraction)	4.4%	3	5.7%	4	0.21
Spontaneo-us vaginal delivery	34.3%	23	28.5%	20	0.14

**(a):** 

Variable	Metformin		Insulin		*P* value
	*N*: 67		*N*: 70		
	%	*N*	%	*N*	
Hypoglycemia	10.4%	7	21.4%	15	0.01
Transient tachypnea	2.9%	2	4.2%	3	0.67
Respiratory distress	1.4%	1	2.8%	2	0.85
Neonatal jaundice	19.4%	14	15.7%	11	0.31
Phototherapy	19.4%	14	15.7%	11	0.31
Neonatal intensive care unit admission	14.9%	10	17.1%	12	0.51
Birth trauma	0%		0%		

**(b):** 

Variable	Metformin		Insulin		*P* value
	*N*: 67		*N*: 70		
	%	*N*	%	*N*	
Apgar score at 5 minutes < 7	1.5%	1	1.4%	1	0.59
Gestational age at birth	38.7 ± 1.1		38.9 ± 1.4		0.06
Estimated weight > 90th percentile	14.9%	10	15.7%	11	0.89
Estimated weight < 10th percentile	5.9%	4	7.1%	5	0.31
Congenital anomalies	1.5%	1	2.8%	2	0.91
